# Arbitrarily high time bandwidth performance in a nonreciprocal optical resonator with broken time invariance

**DOI:** 10.1038/s41598-020-72591-6

**Published:** 2020-09-25

**Authors:** Ivan Cardea, Davide Grassani, Simon J. Fabbri, Jeremy Upham, Robert W. Boyd, Hatice Altug, Sebastian A. Schulz, Kosmas L. Tsakmakidis, Camille-Sophie Brès

**Affiliations:** 1 École Polytechnique Fédérale de Lausanne (EPFL), Photonic Systems Laboratory (PHOSL), 1015 Lausanne, Switzerland; 2 Department of Physics, University of Ottawa, Ottawa, ON Canada; 3 The Institute of Optics, University of Rochester, Rochester, NY 14627 USA; 4 École Polytechnique Fédérale de Lausanne (EPFL), Bionanophotonic Systems Laboratory (BIOS), 1015 Lausanne, Switzerland; 5 School of Physics and Astronomy, SUPA, University of St Andrews, St Andrews, KY169SS UK; 6 Solid State Physics Section, Department of Physics, National and Kapodistrian University of Athens, Panepistimioupolis, 157 84 Athens, Greece; 7 Present Address: Dipartimento di Fisica, Università Degli Studi Di Pavia, via Bassi 6, 27100 Pavia, Italy

**Keywords:** Theoretical physics, Optical physics, Optical physics, Slow light

## Abstract

Most present-day resonant systems, throughout physics and engineering, are characterized by a strict time-reversal symmetry between the rates of energy coupled in and out of the system, which leads to a trade-off between how long a wave can be stored in the system and the system’s bandwidth. Any attempt to reduce the losses of the resonant system, and hence store a (mechanical, acoustic, electronic, optical, or of any other nature) wave for more time, will inevitably also reduce the bandwidth of the system. Until recently, this time-bandwidth limit has been considered fundamental, arising from basic Fourier reciprocity. In this work, using a simple macroscopic, fiber-optic resonator where the nonreciprocity is induced by breaking its time-invariance, we report, in full agreement with accompanying numerical simulations, a time-bandwidth product (TBP) exceeding the ‘fundamental’ limit of ordinary resonant systems by a factor of 30. We show that, although in practice experimental constraints limit our scheme, the TBP can be arbitrarily large, simply dictated by the finesse of the cavity. Our results open the path for designing resonant systems, ubiquitous in physics and engineering, that can simultaneously be broadband and possessing long storage times, thereby offering a potential for new functionalities in wave-matter interactions.

## Introduction

The time-bandwidth product (TBP) is a relational property characterizing all individual resonators, whether they are of mechanical, acoustic, electrical, atomic or optical nature. A general definition of the TBP should consider the product between the *acceptance* bandwidth (∆*ω*_acc_) of the system, which does not necessarily coincide with the measured cavity linewidth as will be explained later, and its characteristic decay, or ‘storage’, time (τ_out_). The majority of present-day resonant systems, are *reciprocal* in nature and, consequently, time-reversal symmetric. Therefore, if wave energy may be coupled out of such systems, an exactly equal amount of energy can be coupled in simply by ‘reversing’ time. In practice, in a reciprocal resonant system, ∆*ω*_acc_ coincides with the cavity linewidth, and, therefore, its TBP is always limited to unity by Fourier relation stating ∆*ω*_cav_ = 1/*τ*_out_, a value commonly referred to as the ‘time-bandwidth limit’^1–3^. This inherent limitation simply dictates that long storage times unavoidably imply narrow input bandwidths, while large bandwidths are unfortunately retained only for short periods of time. Photonics is particularly affected by the time-bandwidth limit. On the one hand, long interaction times are required for storage of optical pulses and efficient light-matter interaction (such as absorption, emission and nonlinear optical effects). On the other hand, broadband signals are desirable since they are normally associated with larger amount of information.

Over the last twenty years, several optical designs aiming at overcoming this limitation have been investigated. One approach consists of leveraging slow-light waveguides. Such systems exploit the characteristic refractive index dispersion near resonances, due to intrinsic electronic transitions^4–6^ or induced by stimulated Brillouin^7,8^ or Raman scattering^9,10^, or Bragg reflections in periodic structures^11^, to slow down the propagation speed of light in the medium. All of these systems operate in the ‘waveguide regime’, even when they include coupled resonator waveguides, where there is single-pass light propagation and continuous dispersion. In this regime, the time-bandwidth performance of the device is inherently different from that of isolated resonators. Rather than being coupled to a resonant mode, light undergoes a *delay* that can be extended by either increasing the group index or the propagation length. Nevertheless, these systems are still characterized in terms of a group-index–bandwidth limit^12^ or a time-delay–bandwidth–footprint limit. In both of these terms, slow-light waveguides are intrinsically limited, and – similarly to resonant systems – the achievable delay times remain inversely proportional to (a power of) the waveguide’s bandwidth, Δ*t* ~ Δ*ω*^−*α*^, where typically *α* = 2 or 3^4,13,14^. Here, the trade-off arises from pulse temporal broadening owing to various dispersion phenomena (2nd and 3rd order dispersion, dispersion of gain/absorption), preventing significant slowing-down of broadband signals^4,7,13,14^.

Another attempt to overcome the time-bandwidth limit was reported some time ago^15^. That scheme made use of temporal *adiabatic* switching of a system between two reciprocal states: a large-bandwidth–short-storage-time state (low quality factor, *Q*, state) and a narrow-bandwidth–long-storage-time one (high-*Q* state). However, while the time-bandwidth limit was marginally exceeded (by a factor of 2 or less), the spectral and temporal shapes of the released pulse were not preserved; rather, they strongly depended on the property of the reopened cavity, leading to substantial distortions of the released pulse^15–20^. Crucially, the simultaneous storage of *multiple* pulses in the system cannot be achieved with that scheme: while the bandwidth of the first pulse is adiabatically compressed, a second pulse cannot be injected into the device.

More recently, a proposal^21^ for arbitrarily overcoming the time-bandwidth limit of *resonant* systems was put forward, based on breaking *Lorentz reciprocity*^22,23^ in the resonant system, without accompanying adiabaticity or signal distortion limitations. This theoretical proposal has reinvigorated a debate about whether (or not) the time-bandwidth limit can be exceeded in resonant systems^24–26^. However, much of this recent theoretical activity on nonreciprocal resonators has been focused on time-invariant systems.

In this work, we provide the first experimental confirmation that inducing nonreciprocity by breaking the time-invariance in a cavity system we can overcome the ‘fundamental’ time-bandwidth limit. Using a macroscopic, fiber-optic resonator, in which Lorentz reciprocity is broken by suitable time modulation (i.e. time variant system), we report a TBP above the fundamental limit of ordinary reciprocal cavities by a factor of 30, solely limited by current experimental constraints of our setup. The *non-adiabatic* switching from fully-open to fully-closed state does not affect the spectral and temporal properties of the injected pulses, and allows for simultaneously storage of multiple pulses. Overall, our resonant system is Lorentz-nonreciprocal owing to breaking of its time-invariance^22,23,27–30^, allowing us to decouple cavity photon lifetime from cavity acceptance bandwidth.

**Figure 1:**
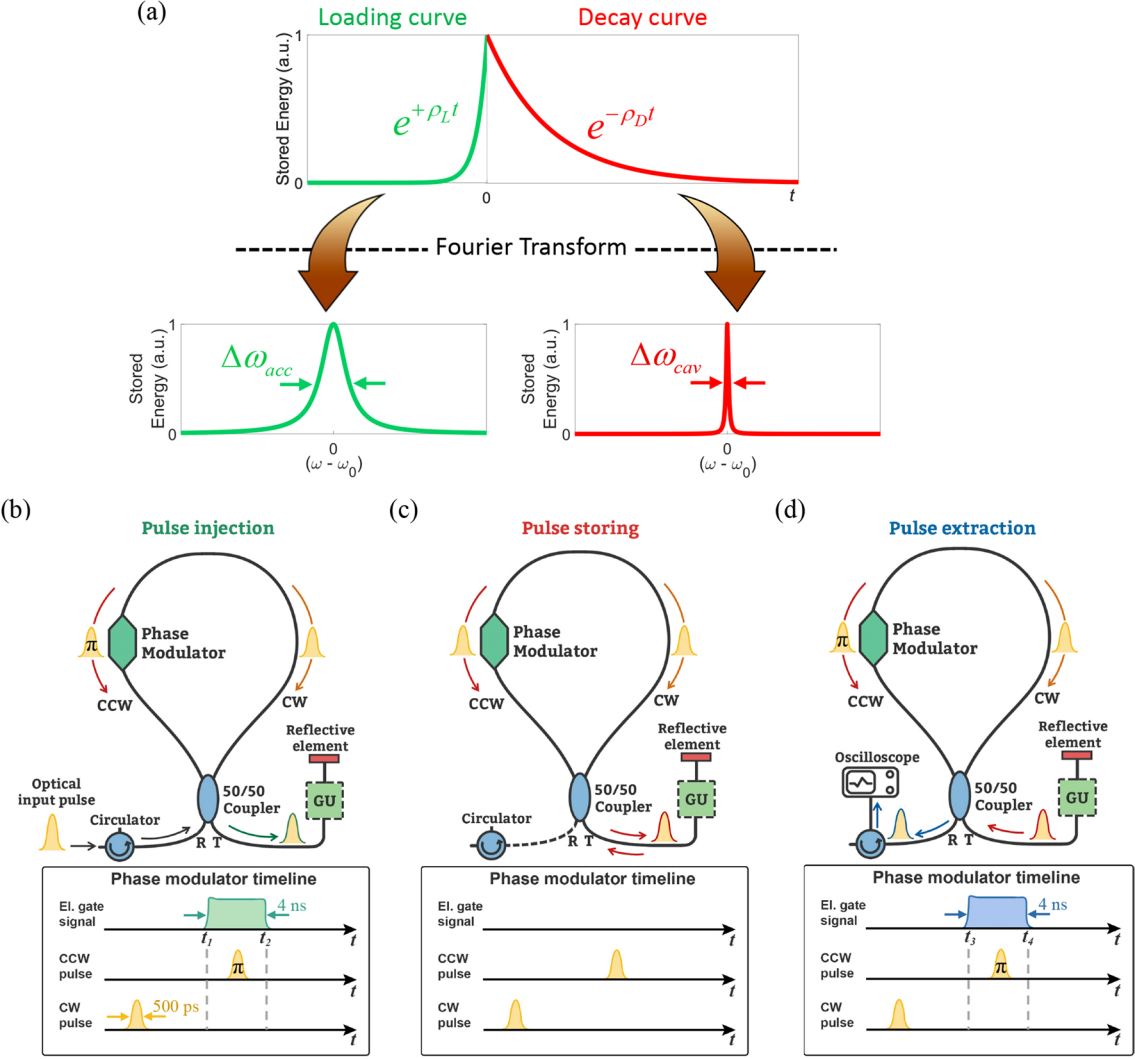
(**a**) Exponential loading and decay curves of a time-variant nonreciprocal resonator. If *ρ*_*L*_ > *ρ*_*D*_, the exponential energy loading process is faster than the decay process, and their associated bandwidths, ∆*ω*_acc_ and ∆*ω*_cav_, respectively, are different, with ∆*ω*_acc_ > ∆*ω*_cav_. Implementation in a Figure-9 resonator: (**b**) Injection—The optical input pulse is fully coupled in the cavity owing to constructive interference of the CW and CCW pulses at the T port when a π phase shift is solely applied to the CCW pulse. (**c**) Storing—Once loaded, if no other gate signal is applied to the modulator, the CW and CCW pulses interfere constructively at the T port and the pulse is stored in the resonator until it is dissipated through internal loss. (**d**) Extraction—The pulse is extracted after a desired number of RTs by opening again the cavity, i.e. applying a second “gate” signal to the phase modulator to the CCW portion of the pulse. A gain unit (GU), can be incorporated to partially compensate for the dissipative loss.

A general definition of the TBP can be obtained in terms of the system’s loading (*ρ*_*L*_) and decay (*ρ*_*D*_) energy rates as:

1 $$TBP = \Delta \omega_{acc} \tau_{out} = \frac{{\Delta \omega_{acc} }}{{\Delta \omega_{cav} }} = \frac{{\rho_{L} }}{{\rho_{D} }}.$$

where ∆*ω*_acc_ and ∆*ω*_cav_, are the full width at half maximum of the Lorentzian functions associated, through the Fourier transform, respectively to the loading and decay curves of the intra-cavity energy (see Supplementary Information). As it is well known, the decay of the energy stored within a cavity is caused by the loss of power through radiative (transmission through coupling elements such as mirrors, couplers etc.) and non-radiative processes (absorption losses), which are taken into account by the out-coupling *ρ*_out_ and intrinsic *ρ*_0_ energy decay rates, respectively. The total decay rate can therefore be expressed as: *ρ*_*D*_ = *ρ*_out_ + *ρ*_0_. Analogously, the loading curve depicts how fast the intra-cavity energy would exponentially grow if the resonator was ‘fed’ through the same processes but reversed in time. As a result, loading rate can be expressed as *ρ*_*L*_ = *ρ*_in_ + *ρ*_0_, with *ρ*_in_ and *ρ*_0_ that now are the in-coupling rate and intrinsic loading rate of energy respectively. In fact, even if the incident light is an arbitrary waveform, the optimum coupling in a resonator is the time reversed version of the decay curve, which corresponds to an exponentially increasing waveform^27,31^. Therefore, the acceptance bandwidth that must be considered is the full width at half maximum (FWHM) of the Lorentzian function obtained from the Fourier transform of the loading curve. It represents the maximum input Lorentzian linewidth allowed by the resonator in one free spectral range (FSR). In reciprocal resonant devices, *ρ*_out_ = *ρ*_in_^21,32^ leading to *ρ*_*L*_ = *ρ*_*D*_. The system is said time-reversal symmetric and, as a result, ∆*ω*_acc_ = ∆*ω*_cav_ and TBP = 1. For such a system, the bandwidth of an incoming pulse must be equal to or smaller than the measured resonance linewidth in order to be entirely coupled in the reciprocal cavity. However, *in a time-variant nonreciprocal system*, we can decouple *ρ*_in_ from *ρ*_out_, so that the time-reversal symmetry no longer holds. If the loading process can be made faster than the decay process, meaning that *ρ*_*L*_ > *ρ*_*D*_, the system can show an arbitrary large TBP. This concept is schematically illustrated in Fig. 1a.

### Experimental implementation

We experimentally implemented such a system, at telecommunication wavelengths (around 1.55 μm), based on a Sagnac interferometer connected to a highly reflective element, also known as Figure-9 fiber cavity^33–36^ (see “Methods”). We use this simple known fiber configuration, similar in some ways to a recirculating fiber loop, as a platform to demonstrate for the first time a corroboration of the theory that a resonant system with a nonreciprocal coupling can exhibit an arbitrarily high TBP^21^. We break the time-invariance by using localized time-varying phase modulation asymmetrically positioned inside the Sagnac loop, thereby inducing nonreciprocity in the overall system (see Supplementary Information). This allows us to change in time the in-coupling/out-coupling energy rate of the resonator, which results in a dynamic control of the cavity *Q*-factor. The induced change is non-adiabatic because the modulation is shorter than the round trip time of the cavity (*T*_RT_), which is the inverse of the frequency separation between the resonance lines^37,38^. As depicted in Fig. 1b, light pulse incident to the R port of the 50/50 coupler is split in clockwise (CW) and counter-clockwise (CCW) pulses travelling through the loop. If no phase modulation takes place, the two pulses travel the exact same path and constructively interfere at the reflection port R, as such exiting the resonator. However, if the phase modulator is electrically gated to shift by *π* the phase of the CCW pulse only, then the pulses constructively interfere at the transmission port T of the coupler and the whole light pulse is directed to the reflective element. During the modulator gating time, say *t*_*1*_ < *t* < *t*_*2*_, the system is thus a *completely open cavity* capable of fully accepting the pulse without any reflection. When the pulse is reflected back into the Sagnac interferometer by the reflective element, if no other gate signal is applied to the modulator, the CW and CCW pulses again interfere constructively at the T port, meaning that the light pulse is trapped (Fig. 1c). The system hereafter, say time *t* > *t*_*2*_, acts as a *completely closed cavity* formed by the Sagnac interferometer and the reflective element. We can extract the pulses from the resonator after a desired number of cavity round trips (RT) by gating once again the phase modulator (for *t*_*2*_ < *t* < *t*_*3*_), leading to switch the constructive interference to the R port, as illustrated in Fig. 1d. It is important to note that during each stage of operation, i.e. injection, storing and release, the system is reciprocal and therefore the acceptance bandwidth coincides with the cavity bandwidth. However, the breaking of time invariance renders the system nonreciprocal^22^, since the system exhibits two different bandwidths during the injection and the storing stages. As any other fiber optic resonator based on standard single mode fiber, this system is subject to the limitations dictated by dispersion and nonlinearity. Specifically, in case of storing of a data pattern made of a sequence of ultra-short pulses, the storage time would be limited by dispersion since the pulses would broaden and could cause the loss of information originally contained in the pattern. This can be dealt, to a certain amount, by dispersion management of the cavity. Besides, an excessively high peak power would induce nonlinear effects, leading to spectral broadening and distortion of the optical bit stream^39^. However, this work does not aim at proposing a novel device, rather at demonstrating a theoretical principle according to which a resonant system with a nonreciprocal coupling can exhibit an arbitrarily high TBP. Therefore, in the experiments, we chose the peak power and the pulse duration in order to have negligible effect of dispersion and nonlinearity, although, in the context of the time-bandwidth performance, in theory, there is no restriction regarding the peak power and the pulse duration.

To express the TBP as a function of the parameters that characterize the Figure-9 resonator, it is convenient to define the energy rates in terms of the in- and out-coupling transmission coefficients of the Sagnac interferometer, *α*_in_ and *α*_out_, respectively. We have: *ρ*_*L*_ = *α*_in_/*T*_RT_ + 1/*τ*_0_ and *ρ*_*D*_ = *α*_out_/*T*_RT_ + 1/*τ*_0,_ (see Supplementary Information), where *τ*_0_ is the internal, non-radiative decay time, usually associated with absorption or energy dissipation inside the cavity. Here *τ*_0_ also takes into account the decay of energy due to the small leakage from the reflective element. When the resonator is in the fully open state at time *t*_*1*_ < *t* < *t*_*2*_, we have *α*_in_ (*t*_1_) = *α*_out_ (*t*_1_) = 1. We can note that the system is actually not a cavity in this case, but an ordinary delay line/waveguide with a reflective termination, and the delay experienced by the pulse is simply *T*_RT_. It thus seems not possible to associate a linewidth to the cavity in the open state. However, as we have already mentioned, the acceptance bandwidth is by definition the FWHM of the Lorentzian profile associated to the energy loading process of the cavity. In this way, a linewidth related to a “fictitious” loading resonant mode, which is quantified by the in-coupling energy rate (*ρ*_in_) and the intrinsic energy rate (*ρ*_0_), can always be associated to the cavity. This is true even in the extreme case of fully open state where *α*_in_ (*t*_1_) = 1, and, therefore, *ρ*_*L*_ (*t*_1_) = 1/*T*_RT_ + 1/*τ*_0_. Once the pulse is coupled into the resonator and the system is switched to the fully closed state at time *t*_2_, we have *α*_in_ (*t*_2_) = *α*_out_ (*t*_2_) = 0 and *ρ*_*D*_ (*t*_2_) = 1/*τ*_0_. Thus, for this time-variant system the TBP reduces to the following simple relation:

2 $$TBP = \frac{{\rho_{L} (t_{1} )}}{{\rho_{D} (t_{2} )}} = \frac{{\tau_{0} }}{{T_{RT} }} + 1 = \frac{{F_{closed} }}{2\pi } + 1$$

with *F*_*closed*_ the finesse of the closed cavity. As a result, by decoupling in time the cavity photon lifetime *τ*_out_ (or equivalently the cavity bandwidth ∆*ω*_cav_) from the cavity acceptance bandwidth ∆*ω*_acc_, such that *ρ*_*L*_ (*t*_1_) > *ρ*_*D*_ (*t*_2_), the TBP of the system can be higher than 1. We stress that, even if the actual bandwidth physically coupled inside the cavity is in practice only limited by the operating frequency region of the 50/50 coupler, the acceptance bandwidth that has to be considered in calculating the TBP is the FWHM of the Lorentzian profile associated to the energy loading process. It is thus not given by the bandwidth of the incoming pulse.

The experimental setup is described in details in the methods. The input to the resonator consists of 500 ps Gaussian optical pulses. Since according to Eq. (2), the cavity finesse limits the TBP, we experimentally control *F*_*closed*_ by inserting a gain unit, which consists in a homemade optical amplifier (EDFA), inside the resonator. As such, we can tune *τ*_0_ by varying the EDFA gain. We measure the cavity RT time to be 48 ns and 120.3 ns, without and with the EDFA respectively. It is important to note that the addition of an EDFA is a means to overcome relatively high absorption losses, adding gain without exceeding the losses, while not affecting the general principle. In fact, an analogous amplification would never increase the TBP beyond one in a reciprocal resonator, as more power would simply also leak out the system at every round trip.

## Results and discussion

**Figure 2:**
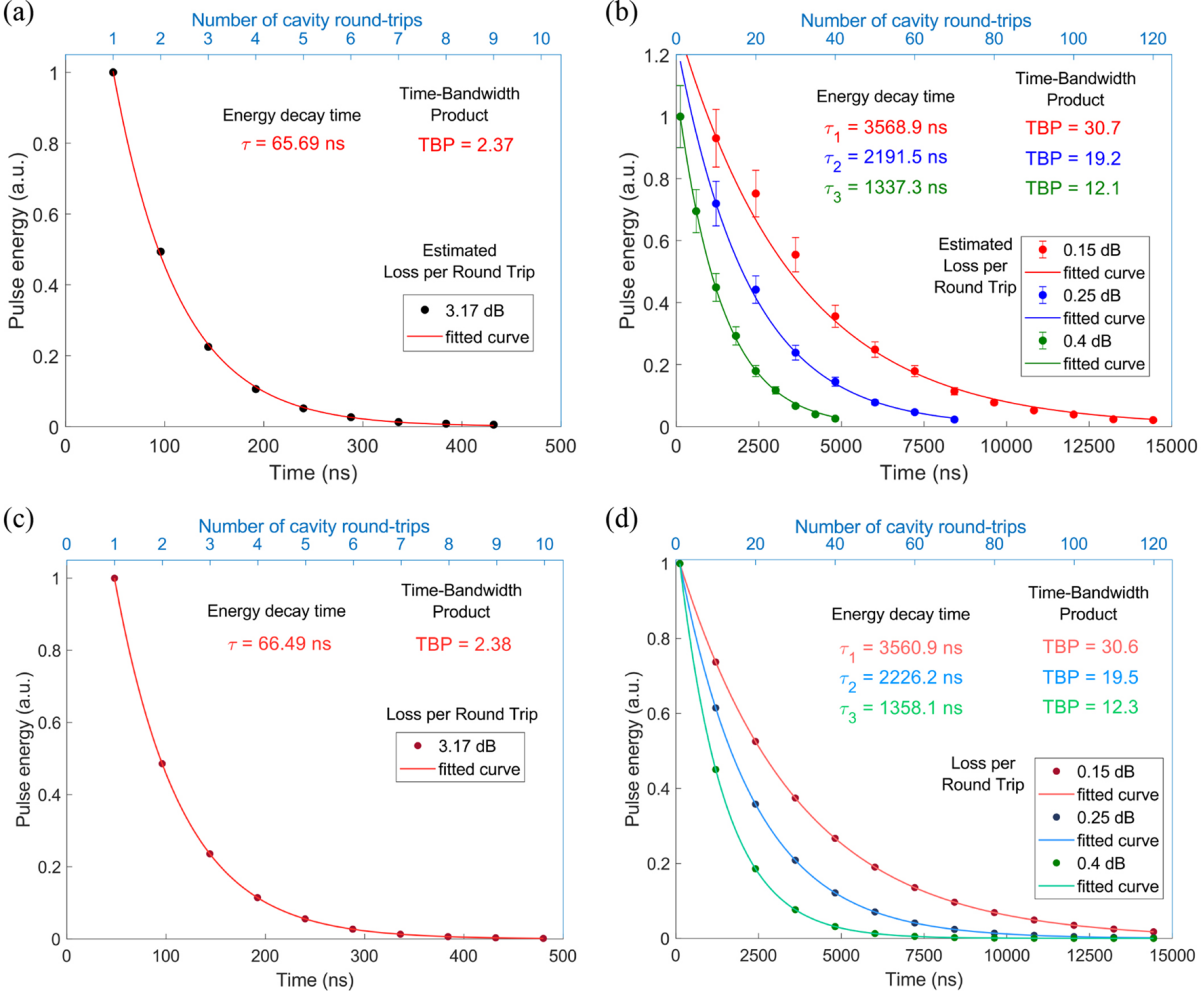
(**a**), Experimentally measured energy decay curve for the 500 ps Gaussian pulse extracted from the full polarization maintaining fiber *passive* resonator at every round trip time (*T*_RT_ = 48 ns). (**b**) Experimentally measured energy decay curves for the pulses extracted from the amplified resonator every ten round trips (with *T*_RT_ = 120.3 ns) for different values of loss per RT. Error bars in (**b**) come from fast polarization rotation due to the non-polarization maintaining erbium doped fiber in the gain unit, resulting in a 20% uncertainty. (**c**) Simulated energy decay curve of the passive cavity with the same actual value of loss/RT as for (**a**). (**d**) Simulated energy decay curves of a passive cavity configuration with the same actual value of loss/RT as for (**b**).

We assess the performance of the system by measuring the energy of the pulse released after different numbers of RTs. Figure 2a shows the result for the passive cavity (no EDFA). The exponential decay fit of the experimental data corresponds to a decay time *τ*_0_ of about 65.69 ns, which allowed us to extract a pulse above the noise level after up to 10 RTs. This corresponds to a closed cavity decay-time of about 1.37 times longer than the cavity RT time, leading to a TBP of 2.37. According to Eq. (2), the maximum achievable TBP can be in principle infinite, providing an infinitely long closed-cavity decay time *τ*_0_, i.e. a loss-less cavity. However, in our case *τ*_0_ is limited by a technological constraint, specifically the absorption losses at the modulator measured to be ~ 3.17 dB/RT. We therefore use the active cavity configuration (with EDFA) to support the claim of arbitrarily large TBP by experimentally controlling the decay time of the system. We progressively adjust the power of the EDFA to partially compensate the intra-cavity loss over three different steps resulting in a net loss of 0.4, 0.25, 0.15 dB/RT. The measurements are shown in Fig. 2b, where the experimental data is normalized to the energy of the pulse extracted after the first cavity RT. As the addition of the EDFA increases *T*_*RT*_, according to Eq. (2), this might actually reduce the TBP of the system. However, the significant increase in *τ*_0_ allows sustaining the pulse for up to 120 RTs (red curve). The decay time strongly increases from 65.69 ns up to 3.57 μs, resulting in a maximum TBP of 30.7. For this measurement, the period of the input pulse train lies between 30 and 31 RTs, to avoid time overlap between the intra-cavity pulse in its 31st round trip and the new incoming input pulse. In this way, we can couple multiple pulses in the resonator and extract an individual pulse after more than 30 RTs without affecting the others.

In principle, we could achieve an even higher TBP value by intensifying the pump power of the EDFA as to fully compensate the round-trip loss. Under these conditions the TBP is higher, but now limited by dispersion, nonlinear effects and the amplification of noise by the EDFA. However, in practice, we were limited by the gain saturation of the doped fiber. This effect can be seen in Fig. 2b for the configuration with 0.15 and 0.25 dB/RT of effective losses. In fact, here the pulses retrieved at the first RT have energies sufficiently high to saturate the gain of the amplifier, which cannot compensate the cavity losses in the same way as for the pulses extracted after more RTs. This results in higher effective cavity losses at the first experimental point, which we therefore excluded from the fit. Further increasing the diode pump power would have affected even more points, misleading the estimate of the TBP.

In order to confirm this concept, we conducted detailed simulations of the pulse storing operation using VPIphotonics software (see “Methods” for details). Our experimental resonator was numerically modelled in 4 passive configurations (without EDFA): in the first one we have reproduced the exact passive experimental cavity, while in the other three configurations we have set the total loss and *T*_RT_ as to mimic the three values of the experimental active setup. The normalized energy of the pulses collected at different RTs is plotted in Fig. 2c for the first case and in Fig. 2d for the other three cases. For all, the TBP value is in excellent agreement with the one calculated after fitting of the experimental data. In particular for Fig. 2d, the exponential decay fits almost perfectly the experiments, showing decay times from 1.36 to 3.56 μs as the dissipative losses progressively decrease. The simulation not only confirm the improvement in TBP but also that we can indeed treat our active cavity as a passive cavity with reduced dissipative losses.

**Figure 3:**
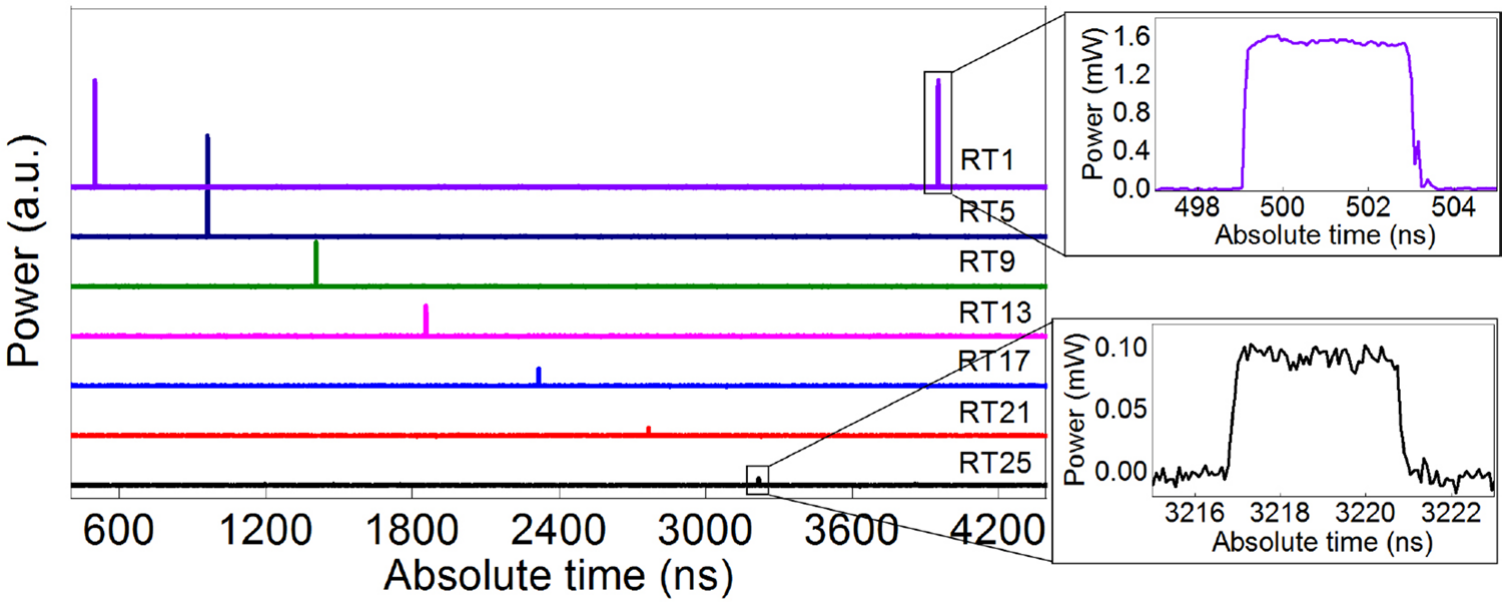
Temporal traces over one period of the optical pulse train extracted after different RTs. The diode pump power of the gain unit was adjusted to obtain a configuration with about 0.5 dB of loss per round trip.

In Fig. 3 we provide an example showing the temporal traces of a 4 ns squared pulse stored in the resonator and extracted after different RTs, with loss of about 0.5 dB/RT. The pulse can be extracted after up to 25 RTs and no leakage is observed between two subsequent extracted pulses. This confirms that we can couple the entire pulse energy (*α*_in_ ≈ 1) without any out-coupling loss (*α*_out_ ≈ 0), switching the cavity from the completely open to the completely closed state. For this specific measurement we used a longer and square-shaped pulse because the acquisition memory of our oscilloscope was not sufficient to detect the 500 ps long Gaussian pulses over the entire time period of the pulse train (about 3.6 μs).

**Figure 4:**
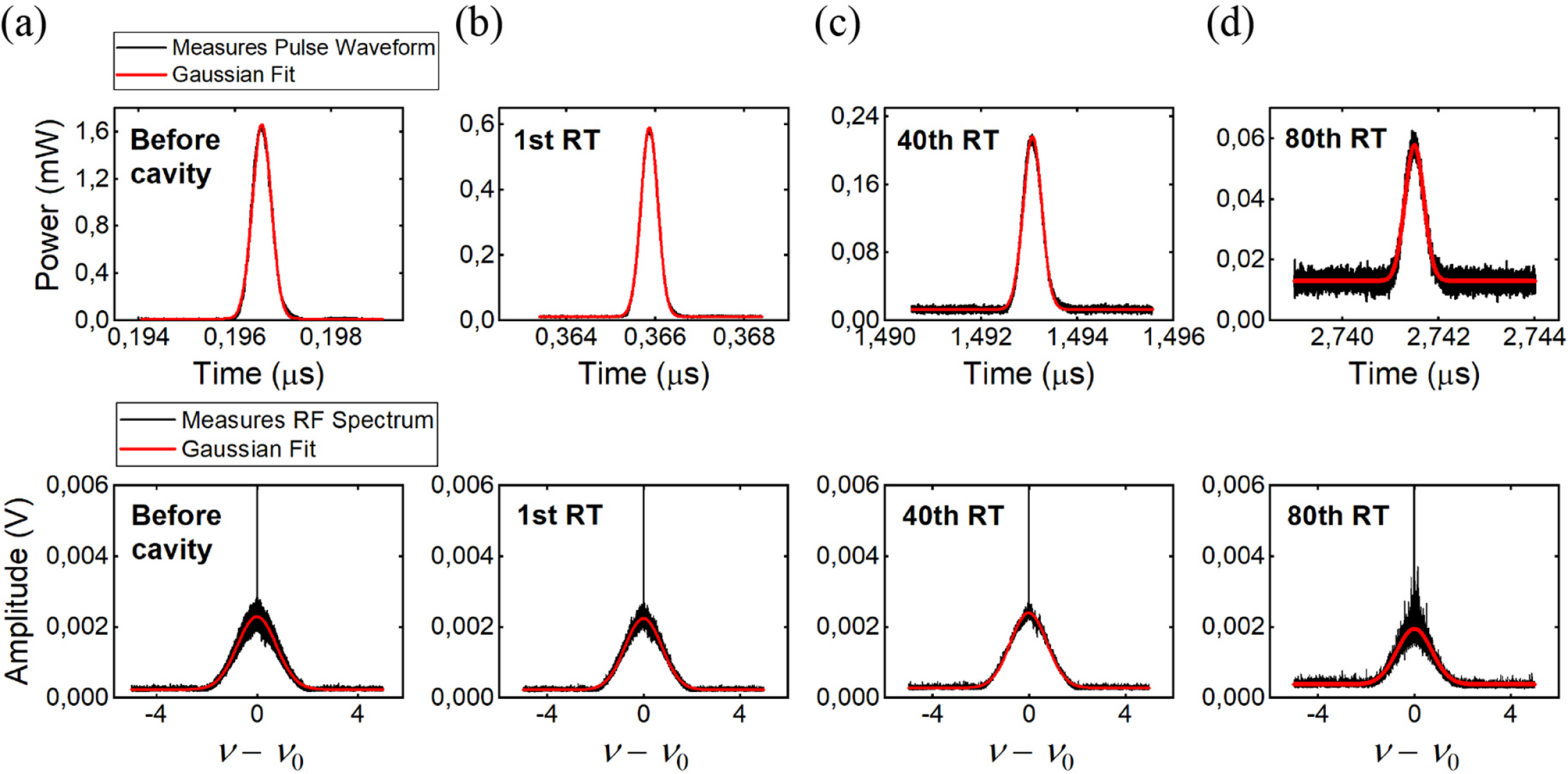
Pulse waveforms and radio-frequency spectra acquired before the cavity (**a**) and after 1 RT (**b**), 40 RTs (**c**) and 80 RTs (**d**). The product of the pulse duration and the bandwidth (FWHM) gives values close to the transform-limited pulse for all the three cases.

Fundamentally different from time-variant devices based on adiabatic tuning^15–20^, here we do not need to adiabatically compress the input pulse bandwidth to match the closed cavity resonance and avoid scattering between different resonant modes. Indeed, we are in a *non-adiabatic* regime, as *T*_RT_ is longer than the tuning time, which is given by the rising time of the phase modulator. Moreover, with *T*_RT_ being longer than the pulse duration, the injected pulse does not interfere with itself and cannot ‘see’ the closed-cavity resonant modes. Therefore, the pulse does not need to adapt to the closed-cavity resonances and, once released, it exhibits a spectrum that is unaffected by the switching between the two different cavity states. To clearly show that the characteristics of the released pulses are preserved over all the RTs, we collected temporal waveforms and radio-frequency (RF) spectra (see “Methods” for details on the measurement technique) of the 500 ps Gaussian pulse after 1, 40 and 80 RTs (Fig. 4b–d) and plotted together with those of the pulse collected before entering the cavity (Fig. 4a). The product of the pulse duration and bandwidth (FWHM) retrieved from the Gaussian fit was always about 0.44 for the investigated RTs, confirming that the pulse does not suffer any measurable distortions.

## Conclusions

In conclusion, we experimentally demonstrated that breaking the time-invariance in a resonant system, thus inducing nonreciprocity, allows to arbitrarily overcome the time-bandwidth limit^21^ by completely decoupling the input energy rate from the cavity decay time. We used localized time-varying phase modulation to dynamically control the *Q*-factor of a macroscopic fiber resonator, which we switched from a completely open to a completely closed state. We proved that the value of the TBP of an individual resonator is ultimately equal to *F*_*closed*_/2*π* + 1 and can be increased at will above the limit, provided that internal, dissipative losses are kept sufficiently low. Mitigating for these dissipative losses with a gain unit, we reported a TBP 30 times above the ‘fundamental’ time-bandwidth limit of ordinary resonators – limited only by current experimental constraints of our setup. Additionally we could simultaneously store and manipulate *multiple* pulses, a key capability missing from previous adiabatic cavity modulation schemes^15,20^. When retrieved, the pulses did not exhibit detectable temporal and spectral distortions. The presented scheme may thus open the path for applications—both fundamental and applied, throughout physics and engineering—where large bandwidths, long storage durations, high sensitivities and strong wave-matter interactions are simultaneously desired^40^.

## Methods

### Description of the experimental setup

The resonator used in the experiments is made of polarization maintaining fibers with a *T*_RT_ of about 48 ns, while the reflective element is a fiber Bragg grating with a center wavelength at 1551.3 nm and a stop bandwidth of about 28.2 GHz. The gain unit is made of 90 cm-long Erbium-doped fiber connected with two fused fiber wavelength division multiplexers and pumped by a semiconductor laser diode at 980 nm. The optical input pulse train at 1551.3 nm is obtained from a laser, modulated in intensity to give 500 ps Gaussian pulses with 894 MHz bandwidth, and a repetition rate corresponding to about 30 cavity RTs. We synchronized an electrical pulse of 4 ns with the optical signal to activate the phase modulator when it is traversed by the CCW pulse only. Once extracted, the pulses are detected at the third port of a circulator, placed before the R port of the Sagnac interferometer, by using a high-speed sampling oscilloscope. Both the electrical signals used to drive the phase and the intensity modulator were generated by the same arbitrary waveform generator (AWG) (Tektronix model 7122B). The phase modulator used for the experiments was a LiNbO_3_ electro-optic modulator (Photline model MPZ-LN-10) with an electro-optic bandwidth of 12 GHz. The synchronization between the electrical “gate” and the optical signal was performed directly from the AWG by imposing a delay on the electrical signal that drove the phase modulator.

### Methods for the measurements of the pulse waveforms and spectra

The temporal traces were registered by detecting the extracted pulses on a sampling oscilloscope with 20 GHz of optical bandwidth. Given the limited resolution of our OSA, a direct measurement of the pulse spectrum in the optical domain did not provide the suitable resolution to detect variations in the spectrum of the order of the cavity free-spectral range (about 8 MHz). We thus implemented a zero-delay self-heterodyne technique^41^, to map the optical spectrum of the pulses into the radio-frequency domain. The pulses retrieved from the resonator were modulated using a 40 GHz Mach–Zehnder modulator to create sidebands at 16 GHz from the central pulse frequency and sent to an Electrical Spectrum Analyzer (ESA). The bottom row of Fig. 4 reports the radio-frequency spectra, given by the convolution of the beating lines acquired with the ESA and centered at the modulation frequency.

### Methods for the numerical simulations

The simulations were performed using the tool VPItransmissionMaker Optical Systems of the software VPIphotonics Design Suite whose numerical solver is based on a full-wave analysis. We reproduced the setup in the graphical environment using built-in blocks with customized parameters.

## Supplementary information

Supplementary file1

## Data Availability

The data that support the findings of this study are available from the corresponding authors on reasonable request.
