# Supplementary material for: Arbitrarily high time bandwidth performance in a nonreciprocal optical resonator with broken time invariance
Source: Sci Rep. 2020 Sep 25;10:15752. doi: 10.1038/s41598-020-72591-6 (PMC7519663; doi:10.1038/s41598-020-72591-6)
Supplement: Supplementary file 1 — Supplementary file1 [file 41598_2020_72591_MOESM1_ESM.docx]

Supplementary Information

**Arbitrarily high time bandwidth performance in a nonreciprocal optical resonator with broken time invariance**

**Ivan Cardea^1^, Davide Grassani^1,2^, Simon J. Fabbri^1^, Jeremy Upham^3^, Robert W. Boyd^3,4^, Hatice Altug^5^, Sebastian A. Schulz^6^, Kosmas L. Tsakmakidis^7^, Camille-Sophie Brès^1*^.**

^1^École Polytechnique Fédérale de Lausanne (EPFL), Photonic Systems Laboratory (PHOSL), Lausanne CH-1015, Switzerland.

^2^Currently with Dipartimento di Fisica, Università degli studi di Pavia, via Bassi 6, 27100 Pavia, Italy.

^3^Department of Physics, University of Ottawa, Ottawa, ON, Canada.

^4^The Institute of Optics, University of Rochester, Rochester, New York 14627, USA

^5^École Polytechnique Fédérale de Lausanne (EPFL), Bionanophotonic Systems Laboratory (BIOS), Lausanne CH-1015, Switzerland.

^6^School of Physics and Astronomy, SUPA, University of St Andrews, St Andrews, KY169SS, UK.

^7^Solid State Physics section, Department of Physics, National and Kapodistrian University of Athens, Panepistimioupolis, GR - 157 84, Athens, Greece.

*Correspondence to: [camille.bres@epfl.ch](mailto:camille.bres@epfl.ch).

# Acceptance bandwidth and time-bandwidth product (TBP) in reciprocal and non-reciprocal time-variant resonant systems

The time-bandwidth product (TBP) of a resonator, or cavity, is more generally defined as the product between its acceptance bandwidth (Δ*ω*_acc_) and its characteristic decay time (*τ*_out_). This very general definition keeps the concept of the bandwidth that the resonator can “accept” separated from what is considered as the cavity bandwidth (Δ*ω*_cav_), usually defined as the inverse of *τ*_out_. In fact, as we will show here, these two quantities can be different and completely decoupled. This becomes clear considering the temporal evolution of the energy stored in the cavity and comparing it with the same process reversed in time, to which hereafter we will refer as *loading* process. As illustrated in Figure S1, the energy stored in a resonator at *t* =0 is |*a_D_* (0)|^2^, and decays exponentially as *e^−^*^(^*^D^*^)^*^t^* (red curve), where *ρ_D_* = *ρ*_out_ + *ρ*_0_ is the energy decay rate due both to non-radiative and radiative processes, which are related to internal absorption and out-coupling from the cavity respectively. The Fourier transform of such exponential decay curve is a Lorentzian function, whose FWHM is just *ρ_D_*, which is commonly identified as the cavity bandwidth (i.e. Δ*ω*_cav_ = *ρ_D_*). Consider the energy inside the resonator at the time *t* = *T*, that is|*a_D_* (*T*)|^2^, as the final state of the decay process. Then, let’s apply the time-reversal operation, which mathematically corresponds to reversing the sign of the time variable in the decay process^1–3^. Doing so, |*a_D_* (*T*)|^2^ becomes the initial state of the loading process at the time *t* = −*T*, that is |*a_D_* (*T*)|^2^ = |*a_L_* (−*T*)|^2^. Now, the energy flows and builds up in the resonator as *e*^+(^*^L^*^)^*^t^*, where *ρ_L_* = *ρ*_in_ + *ρ*_0_ is the energy *loading rate*. If *ρ_L_* is equal to *ρ_D_* the time-reversal symmetry holds (orange-dashed curve in Fig S1). However, if they are different, owing to an asymmetry of the radiative process (*ρ*_in_ ≠ *ρ*_out_), the time-reversal symmetry is broken leading to nonreciprocity in the system. Particularly, if *ρ*_in_ *> *_out_, after a time *T*, the energy in the resonator would be higher than the initial value of the energy in the decay process (|*a_L_* (0)|^2^ > |*a_D_* (0)|^2^ − green-dashed curve in Figure S1)*.* It should be noticed that, this latter scenario require the breaking of one of the two hypothesis on which reciprocal resonant systems are based, i.e. linearity or time-invariance^1,4^.

In the same way as in the energy decaying process, through the Fourier transform, we can associate a bandwidth to this *loading* process given by the FWHM of the corresponding Lorentzian function, which in this case is equal to *ρ_L_*, and represents the acceptance bandwidth of the resonator. In fact, being *a_L_* (*t*) the field inside the resonator, we can write:

where *u*(*t*) is the Heaviside step function and *ω_0_* is the resonant frequency. The corresponding Fourier transform is:


.

The expression of the energy inside the cavity is obtained by taking the square modulus of *a_L_* (*t*):

 .

The loading process is thus characterized by a Lorentzian function too, and its FWHM represents the acceptance bandwidth of the resonator:

In reciprocal resonant systems, *ρ*_in_= *ρ*_out_, therefore *ρ_L_* = *ρ_D_* and Δ*ω*_acc_ = Δ*ω*_cav_. The TBP of such reciprocal system (*TBP_R_*) is equal to 1:

However, if *ρ*_in_≠ *ρ*_out_, the resonant system is nonreciprocal and, if *ρ*_in_ > *ρ*_out_, the loading process reaches a final state at *t* =0 that is higher than the initial state of the decaying process (see Fig. S1). Therefore, the acceptance bandwidth results to be larger than the cavity bandwidth, leading to a TBP for such nonreciprocal system (*TBP_NR_*) given by:

In the Figure-9 resonator the nonreciprocity is obtained by breaking the time-invariance of the system. As explained in Figure S2a, either in the open and the closed state the resonator exhibits identical in- and out-coupling energy rate (*ρ*_in-open_ = *ρ*_out-open_ and *ρ*_in-closed_ = *ρ*_out-closed_). However, *ρ*_in-open_ > *ρ*_out-closed_. As a result, the resonator can be filled with energy at a rate *ρ_L_*_-open_, but once the system is switched to the closed state, the energy decay occurs at a rate *ρ_D_*_-closed_ < *ρ_L_*_-open_ as illustrated in Figure S2b. Therefore, although the resonant system is reciprocal at any given time since its time-reversal symmetry is never broken, it can be considered nonreciprocal given the temporal switching between the open and closed states. This nonreciprocity induced by the broken time-invariance of the system leads to a decoupling of the acceptance bandwidth from the characteristic decay rate of the cavity, allowing to couple energy in the system at a rate higher than the value imposed by the cavity bandwidth^5^. We stress that, the exponentially increasing loading curve represents the optimum coupling of a resonator, not the actual coupling of energy within the cavity, which can take any form in time domain and occur only through radiative processes, since the internal (non-radiative) absorption is an irreversible process.

# Transmission and reflection coefficients of the Figure-9 resonator

In general, the Figure-9 cavity is formed by a Sagnac interferometer, or fiber loop mirror, connected to a highly reflecting element (see left part of Figure S3). For the sake of simplicity, the structure of the Figure-9 cavity can be seen as a Fabry-Pérot cavity, as shown in Figure S3, in which the fiber loop mirror represents one of the two reflecting elements with reflection, transmission and attenuation given by those of the Sagnac interferometer. The fiber optic system used in the experiments was based on such a cavity using a high reflectivity fiber Bragg grating (FBG) connected to one of the input ports of a fiber loop mirror (port T in Figure S3). The bandwidth of the FBG was chosen to be large enough to cover the pulse bandwidth, but sufficiently narrow to filter out the amplified spontaneous emission generated by the doped fiber, which, otherwise, would have overwhelmed the signal after few cavity round trips, dramatically reducing the signal-to-noise ratio.

The reflection and transmission coefficients of the fiber loop mirror can be found considering a monochromatic light wave (*E*_in_) of frequency *ω* incident on the port R of the directional coupler, as schematically illustrated in Figure S3. The amplitude of the wave is split by the coupler in two parts that travel through the same physical path in opposite direction to recombine again in the coupler. Assuming that there is no relative phase delay between the two counter-propagating fields and, recalling that light coupled across the coupler suffer a *π*/2 phase lag with respect to light travelling straight through it, we can write the complex amplitudes of the reflected and transmitted fields as following:

where *κ* and *τ* are the cross and straight-through field coupling coefficient of the coupler. The losses in the Sagnac interferometer (*α*) give the attenuation factor (*a*_p_ = *e^−αL^*), while *φ*_p_ is the phase delay experienced by the fields during the propagation between the ports 1 and 2 of the coupler.

The *field* reflection and transmission coefficients of the fiber loop are easily found from the above equations:

 .

The corresponding *power* reflection and transmission coefficients are obtained by taking the square modulus of *r*_FL_ and *t*_FL_:

for which the following relation holds: |*t*_FL_|^2^ + |*r*_FL_|^2^ = *a*_p_.

In our experiment, we used a 50/50 coupler, that is *κ^2^*= *τ^2^*= 0.5. Inserting these values in the equation (S10), in absence of any phase difference between the two waves in the Sagnac interferometer, the incident field is totally reflected due to the constructive interference at the R port of the coupler, while the transmission at the T port of the fiber loop mirror is zero (|*r*_FL_|^2^ = *a*_p_ and |*t*_FL_|^2^ = 0). In this configuration, the cavity can be considered completely “closed”, because the field incident upon it is totally reflected, apart from an attenuation factor.

If there is a relative phase difference equal to *π* between the two counter-propagating fields, from equations (S7) and (S8) we get:

and assuming an ideal coupler (*τ^^* +*κ^^* =  we obtain:

.

Thus, the incident field is totally transmitted (and partially attenuated) through the fiber loop mirror due to the constructive interference at the port T of the coupler (regardless the value of the coupling coefficient). In this state the cavity is then completely “open”.

# Energy rate coefficients of the time-variant Figure-9 resonator

In the reported experiments, the localized phase variation was provided by an electrically-driven phase modulator, placed at an offset position from the loop midpoint to ensure that the phase shift was imparted only to one of the two counter-propagating pulses. In this way we were able to change in time the reflection and transmission coefficients of the fiber loop, similarly to the mechanism used to switch optical pulses in Terahertz optical asymmetric demultiplexers (TOADs)^6^. In the analogy previously used, the fiber loop represents the front mirror of the Fabry-Pérot resonator, and we use the time-variant phase modulation to dynamically control the *Q*-factor of the cavity. In our system, the pulse duration is smaller than the cavity round trip time and we can entirely inject the incoming pulse within the cavity, by driving the modulator using an electrical (“gate”) signal of appropriate amplitude and duration at least equal to the one of the optical pulse. The “gate” is synchronized with the counter-clockwise (CCW) pulse. The pulse switched to the port T of the Sagnac interferometer is reflected by the FBG and travels again through the fiber loop mirror. At this point, no other phase shift is applied and the pulse bounces back and forth between the fiber loop and the FBG until it is extracted after a desired number of round trips applying a second electrical “gate”. The pulse train is designed such that a given pulse coupled into the cavity does not overlap, inside the phase shifting element, with the subsequent pulse. As a result, the pulse entering the resonator experiences the power coefficients of the cavity in the open state (|*r*_FL_|^2^ = 0 and |*t*_FL_|^2^ = *a*_p_), while the power coefficients seen by the pulse already stored in the resonator are those of the cavity in the closed state (|*r*_FL_|^2^ = *a*_p_ and |*t*_FL_|^2^ = 0). The resonator operating in this way can be considered nonreciprocal since it exhibits unequal in-coupling and out-coupling energy rates respectively before and after the energy has been coupled in the cavity. These energy rates results to be given by the transmission coefficients of the fiber loop mirror relative to the cavity in the open and closed state respectively, divided by the cavity round trip time:

 .

In equation (S14), the arrows in the power transmission coefficients have been added to indicate the two different states: from left to right for the transmission coefficient in the open state given by |*t*_FL_|^2^_→_ = (*τ*^^ +*κ*^^^^*a*_p_ = *a*_p_; and from right to left for the transmission coefficient in the closed state, which results to be |*t*_FL_|^2^_←_ = (*τ*^^ −*κ*^^^^*a*_p_.

It should be noticed that, by modelling the Figure-9 cavity as a Fabry-Pérot cavity, we treat the Sagnac interferometer as a lossy mirror with attenuation factor *a*_p_. However, the fiber loop mirror is itself part of the Figure-9 cavity, therefore its losses become part of the attenuation experienced by the pulse in one round trip. Therefore, if we want to find the in-coupling and out-coupling energy rates of the Figure-9 resonator, that correspond to the energy rates during the loading and decay process respectively, we have to divide the coefficients in equation (S14) by the attenuation factor *a*_p._

where *α*_in_ = (*τ*^^ +*κ*^^^^ and *α*_out_ = (*τ*^^ −*κ*^^^^ represent the transmission coefficients of the input port of the Figure-9 resonator.

Note that, assuming an ideal coupler (*τ^^* +*κ^^* =  *α*_in_ is always equal to 1 regardless the value of the field coupling coefficients *τ* and *κ* of the coupler, while *α*_out_ can range from 0 to 1 according to the values of *τ* and *κ*.

By summing the coupling energy rates of the Figure-9 resonator and the energy rates due to the internal (non-radiative) losses, we obtain the loading and decay rates associated respectively to the loading and decay process used for the calculation of the TBP:

# References

1. Caloz, C. *et al.* Electromagnetic Nonreciprocity. *Phys. Rev. Appl.* **10**, 047001 (2018).

2. Leuchs, G. & Sondermann, M. Time-reversal symmetry in optics. *Phys. Scr.* **85**, 058101 (2012).

3. Heugel, S., Villar, A. S., Sondermann, M., Peschel, U. & Leuchs, G. On the analogy between a single atom and an optical resonator. *Laser Phys.* **20**, 100–106 (2010).

4. Mann, S. A., Sounas, D. L. & Alù, A. Nonreciprocal Cavities and the Time-Bandwidth Limit. *Optica* **6**, 104–110 (2019).

5. Tsakmakidis, K. L. *et al.* Breaking Lorentz reciprocity to overcome the time-bandwidth limit in physics and engineering. *Science* **356**, 1260–1264 (2017).

6. Sokoloff, J. P., Prucnal, P. R., Glesk, I. & Kane, M. A Terahertz Optical Asymmetric Demultiplexer (TOAD). *IEEE Photonics Technol. Lett.* **5**, 787–790 (1993).

**Figures**


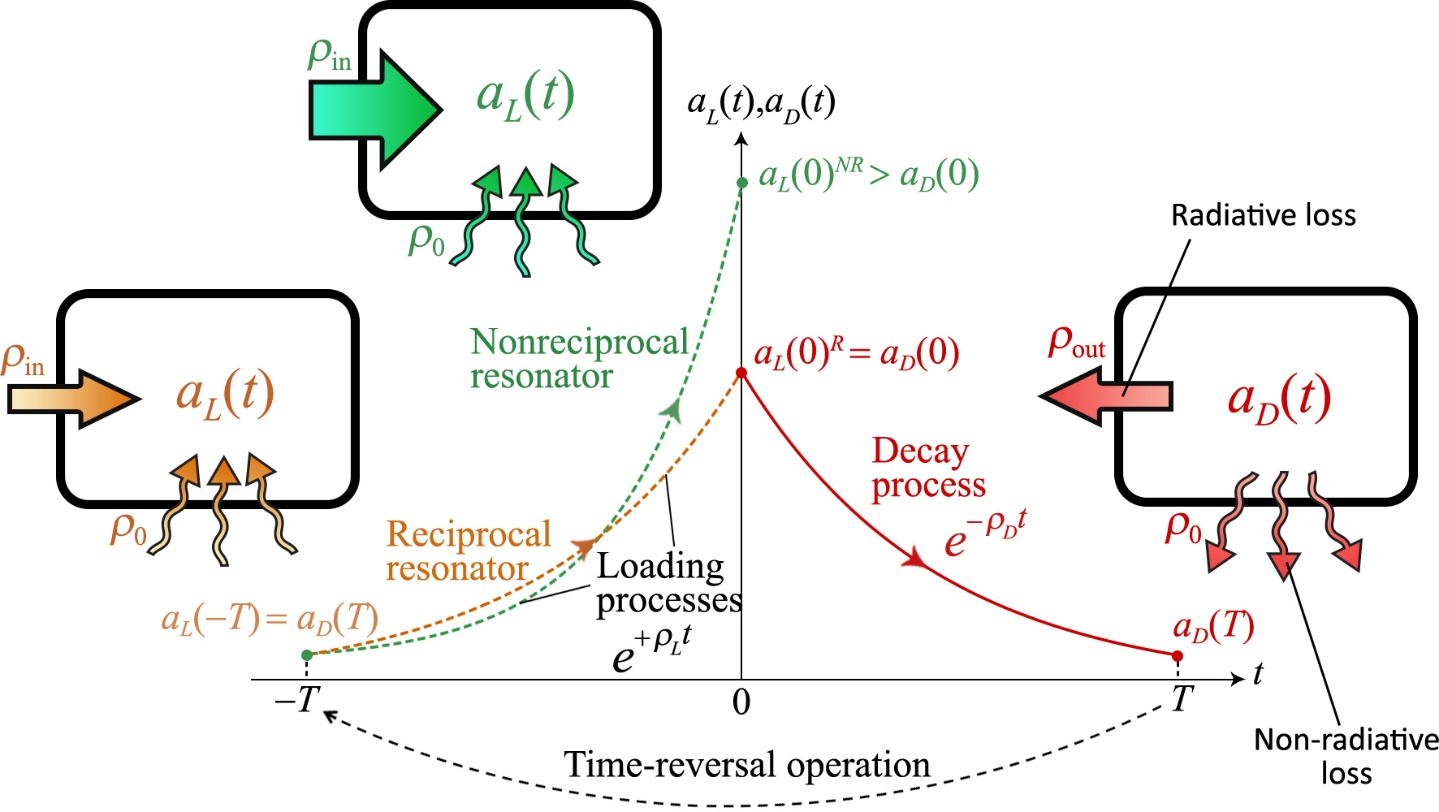


**Figure S1.** Graph of the time-reversal operation of the energy decay process for reciprocal and non-reciprocal resonant systems and their illustrative representations. The decay of the energy stored within the cavity (red curve) is due to the loss of power through radiative (transmission through coupling elements such as mirrors, couplers etc.) and non-radiative processes (absorption and scattering losses), which are taken into account by the out-coupling *ρ*_out_ and intrinsic *ρ*_0_ energy rate, respectively (*ρ_D_* = *ρ*_out_ + *ρ*_0_). The loading curves (green- and orange-dashed curves) depicts how fast the intra-cavity energy would exponentially grow if the resonator was ‘fed’ through the same processes reversed in time, with the radiative and non-radiative processes that becomes the in-coupling energy rate and intrinsic loading rate of energy respectively (*ρ_L_* = *ρ*_in_ + *ρ*_0_). If the resonant system is reciprocal, the decay process and its corresponding time-reversed process are identical. However, if the in-coupling energy rate is higher than the out-coupling energy rate, the exponential energy decay and its corresponding time-reversed process (energy loading) are different and the intra-cavity energy reaches a value at the final state greater than the initial energy state of the decay process. In this case the system is said time-reversal asymmetric, hence non-reciprocal^1^.


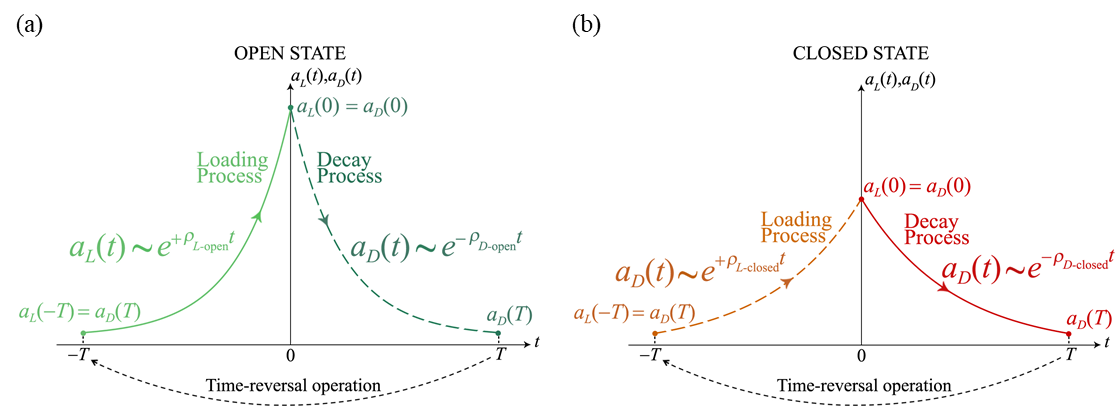


**Figure S2.** Loading and decay processes of a time-variant resonator. In the open state (**a**) and in the closed state (**b**) the energy in the resonant system builds up and decays at the same rate: *ρ_L_*_-open_* *= *ρ_D_*_-open_ and *ρ_L_*_-closed_* *= *ρ_D_*_-closed_ respectively. The time-reversal symmetry is never broken during each state, therefore the system results to be reciprocal at any given time. Nevertheless, the resonator is nonreciprocal owing to a breaking of its time-invariance since it switches from the open to the closed state after energy has been coupled in the resonator in a time period shorter than the round-trip time. The solid lines in (**a**) and (**b**), represent the loading and decay process, respectively, during the open and closed state of the nonreciprocal time-variant resonator before and after the switching respectively.


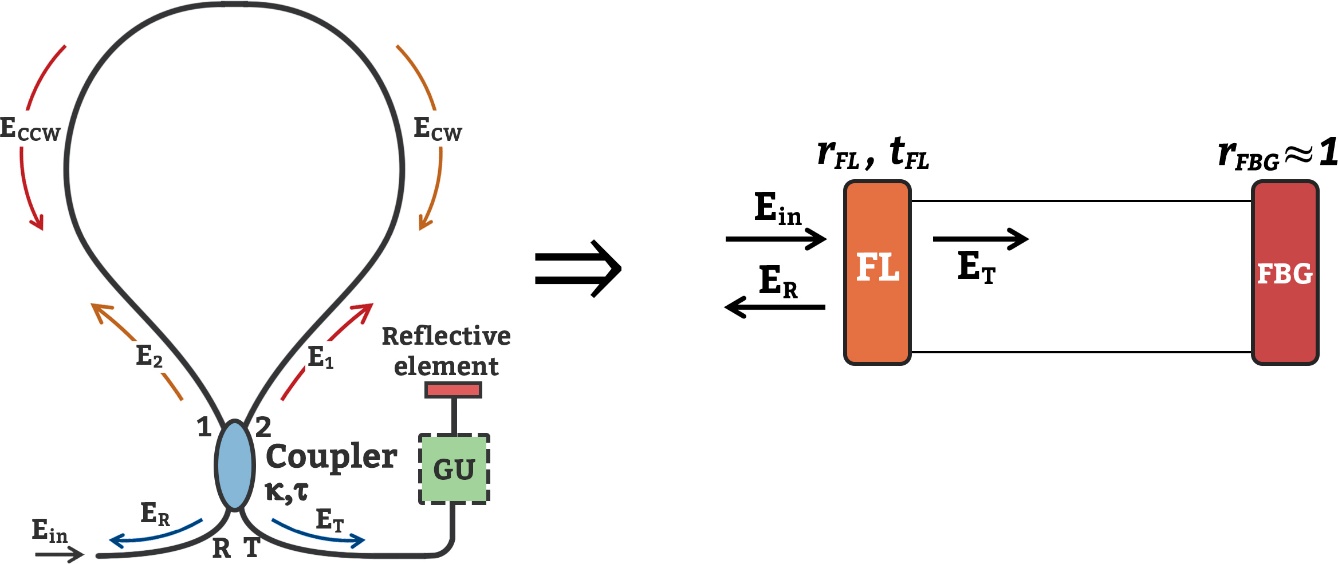


**Figure S3.** Basic scheme of the Figure-9 resonator (left) and the equivalent layout of a Fabry-Pérot cavity (right), where FL indicates the Sagnac interferometer (also known as a fiber loop mirror), and FBG the fiber Bragg grating.
